# Cortical Processing of Multimodal Sensory Learning in Human Neonates

**DOI:** 10.1093/cercor/bhaa340

**Published:** 2020-11-18

**Authors:** S Dall'Orso, W P Fifer, P D Balsam, J Brandon, C O’Keefe, T Poppe, K Vecchiato, A D Edwards, E Burdet, T Arichi

**Affiliations:** Department of Bioengineering, Imperial College London, London SW7 2AZ, UK; Centre for the Developing Brain, School of Biomedical Engineering and Imaging Sciences, Kings College London, London SE1 7EH, UK; Department of Electrical Engineering, Chalmers University of Technology, Gothenburg 412 96, Sweden; Department of Psychiatry, Columbia University, New York 10032, NY; Department of Psychiatry, Columbia University, New York 10032, NY; Centre for the Developing Brain, School of Biomedical Engineering and Imaging Sciences, Kings College London, London SE1 7EH, UK; Centre for the Developing Brain, School of Biomedical Engineering and Imaging Sciences, Kings College London, London SE1 7EH, UK; Centre for the Developing Brain, School of Biomedical Engineering and Imaging Sciences, Kings College London, London SE1 7EH, UK; Centre for the Developing Brain, School of Biomedical Engineering and Imaging Sciences, Kings College London, London SE1 7EH, UK; Department of Bioengineering, Imperial College London, London SW7 2AZ, UK; Centre for the Developing Brain, School of Biomedical Engineering and Imaging Sciences, Kings College London, London SE1 7EH, UK; Department of Bioengineering, Imperial College London, London SW7 2AZ, UK; Department of Bioengineering, Imperial College London, London SW7 2AZ, UK; Centre for the Developing Brain, School of Biomedical Engineering and Imaging Sciences, Kings College London, London SE1 7EH, UK; Paediatric Neurosciences, Evelina London Children’s Hospital, St Thomas’ Hospital, London SE1 7EH, UK

**Keywords:** brain plasticity, classical conditioning, functional MRI, multisensory integration, neonate

## Abstract

Following birth, infants must immediately process and rapidly adapt to the array of unknown sensory experiences associated with their new ex-utero environment. However, although it is known that unimodal stimuli induce activity in the corresponding primary sensory cortices of the newborn brain, it is unclear how multimodal stimuli are processed and integrated across modalities. The latter is essential for learning and understanding environmental contingencies through encoding relationships between sensory experiences; and ultimately likely subserves development of life-long skills such as speech and language. Here, for the first time, we map the intracerebral processing which underlies auditory-sensorimotor classical conditioning in a group of 13 neonates (median gestational age at birth: 38 weeks + 4 days, range: 32 weeks + 2 days to 41 weeks + 6 days; median postmenstrual age at scan: 40 weeks + 5 days, range: 38 weeks + 3 days to 42 weeks + 1 days) with blood-oxygen-level-dependent (BOLD) functional magnetic resonance imaging (MRI) and magnetic resonance (MR) compatible robotics. We demonstrate that classical conditioning can induce crossmodal changes within putative unimodal sensory cortex even in the absence of its archetypal substrate. Our results also suggest that multimodal learning is associated with network wide activity within the conditioned neural system. These findings suggest that in early life, external multimodal sensory stimulation and integration shapes activity in the developing cortex and may influence its associated functional network architecture.

## Introduction

Identifying the neural substrates of learning is critical to understand how experiences are stored in the brain and can be used to form a meaningful representation of the external world. These processes are particularly important for newborn infants, who immediately following birth are exposed to a dramatic change in their environment and consequently must be capable of rapidly adapting their behavior to cope with a wealth of new external experiences. An additional challenge of any natural environment is that meaningful information is often multimodal in origin. Consequently, the newborn nervous system must also quickly adapt to process the temporal, spatial, and contextual features of complex inputs which simultaneously stimulate different sensory systems. This integration between different sensory modalities is thought to be established through associative learning during postnatal life as it allows simple and fast encoding of environmental contingencies (Wallace 2004; Connolly 2014). Consistent with this, it has been found to have multiple roles in early development such as: a prominent function in early life survival through anticipating and overcoming respiratory or thermal challenges during sleep (Paluszynska et al. 2004), facilitating parent–child bonding (DeCasper and Fifer 1980), behavioral and emotional-self-regulation (Lipsitt 1998), integrating attention to facial and vocal expressions (Walker-Andrews 1997), linking different auditory–visual features to make inferences about specific objects (Morrongiello et al. 1998), and facilitating development of language and vocabulary growth (Smith and Yu 2008; Yu 2008).

The basic principles of associative learning can be investigated using classical conditioning, in which a conditioned response (CR) to a specific conditioning stimulus (CS) is learnt through repeated pairing of the CS and another distinct unconditioned stimulus (US). This approach has been successfully employed to model the nature of associative learning by studying experience-dependent changes of behavior and brain activity in animals (Li et al. 2003) and adult humans (Ramnani et al. 2000). Human infants within the first few days after birth have also been shown to be capable of learning the association between odor and tactile stimuli (Sullivan et al. 1991) and between an auditory tone and a mild puff of air to the eyelid (Fifer et al. 2010). However, although such classical conditioning induced behavioral changes can be readily identified in infants, it is unclear whether the resulting motor responses arose from brainstem mediated reflexive activity alone or involved processing at a higher cortical level. Unpacking this distinction is crucial, as it would not only inform about the neural processes which underlie this fundamental ability but importantly also about how early experience can shape patterns of brain activity in the developing brain.

In addition, it is unclear whether hierarchical processes form the basis of multisensory integration in the developing cortex, that is, if information from different sensory systems is first processed in the relevant unimodal sensory cortex and later integrated at a higher level; or if the primary sensory cortices themselves act as multisensory processors which are capable of directly encoding the association (Connolly 2014). In keeping with the latter, emerging evidence suggests that the primary sensory cortices are not merely sensory testers, but are involved in multisensory integration and high-level processes such as memory encoding (Foxe et al. 2002; Murray et al. 2005; Schroeder and Foxe 2005; Watkins et al. 2006; Headley and Weinberger 2015). We therefore hypothesized that the behavioral evidence of associative learning in neonates is governed by underlying intracerebral processes; and that the primary sensory cortices play a critical role in both multisensory integration and encoding of the association between related stimuli even across sensory modalities.

Noninvasive neuroimaging methods and in particular functional magnetic resonance imaging (fMRI) can potentially address these open questions, although challenges inherent in performing these kinds of studies in infants (such as a lack of suitable and safe stimuli, and reduced compliance resulting in decreased tolerance and uncontrolled head motion) have previously precluded successful implementation.

**Figure 1:**
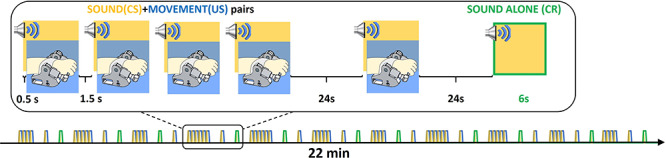
Schematic of the associative learning paradigm. Yellow areas depict the occurrence of the sound (6 s, CS), blue the occurrence of the passive movement (US), and highlighted in green a CR trial in which the sound was played alone. Blocks of paired sound and passive movement (starting with 500 ms lag and coterminating) are repeated a variable number of times (4–6) following two trials: one coupled and one sound alone (CR). The paradigm comprised 11 CR trials lasting 22 minutes in total.

To test our hypotheses, we developed a classical conditioning paradigm to characterize the underlying brain activity during learning using fMRI in a group of naturally sleeping neonates. To understand the developmental context of any identified patterns of brain activity and determine whether they were exclusive to the infant period or represent fundamental processes that continue throughout life, we also presented the same paradigm to a group of healthy adult volunteers. This paradigm consisted of an auditory stimulus and a precisely controlled sensorimotor stimulus (flexion/extension of the right wrist) generated by a custom-built MR compatible robotic device (Allievi et al. 2013). The auditory and sensorimotor systems were selected as substrates for this experiment as there is known to be well-established integration between these systems in the adult brain, which subserves key skills emerging in early childhood including speech production and music perception (Zatorre et al. 2007; Shiller et al. 2010; Provasi et al. 2014). Associative learning was induced using a delay classical conditioning paradigm consisting of repeated blocks of the two stimulus types presented together: with the auditory stimulus as the CS and passive movement as the coterminating US. The CR following learning was then assessed by identifying the functional response to an interleaved auditory alone test condition (see Fig. 1 and Methods section).

## Methods

The study was approved by the NHS research ethics committee (REC code: 12/LO/1247) and informed written consent was obtained from each subject or one parent prior to participation.

### Study Population

The study population consisted of 24 healthy infants studied at term equivalent age (median gestational age at birth: 38 weeks + 4 days, range: 30 weeks + 4 days to 40 weeks + 4 days; scanned at term equivalent age: median post menstrual age 40 weeks + 6 days, range: 38 weeks + 2 days to 42 weeks + 3 days) who were recruited from the Neonatal Intensive Care Unit (NICU) or postnatal wards of St Thomas’ Hospital, London, UK. Of those infants, 20 received a learning paradigm and 4 received a control procedure. All infants were screened by reviewing their antenatal, birth, and clinical histories (including any prior neuroimaging if available) to ensure that they were entirely healthy and representative of the neonatal population. All were clinically stable at the time of scanning (as assessed by an experienced pediatrician), had passed their UK newborn hearing screening (otoacoustic emission and auditory evoked brainstem response tests), and did not require any respiratory support during data acquisition. Infants were excluded from the study group if they were known to have a neurological disease or severe brain injury such as extensive intraventricular hemorrhage (grade 3 with ventricular dilatation or grade 4 with parenchymal extension) or stroke, a diagnosed congenital brain abnormality, and/or a clinical history of birth asphyxia or neonatal encephalopathy. The adult study group consisted of 10 healthy adult volunteers (6 females, 4 males, age range: 25–39-years old).

### Data Acquisition

All infants were studied during natural sleep following feeding, by swaddling in a blanket and then immobilizing using a vacuum evacuated bag (Med-Vac, CFI Medical Solutions, Fenton, MI, USA). Hearing protection using molded dental putty in the external auditory meatus (President Putty, Coltene Whaledent, Mahwah, NJ, USA) and adhesive earmuffs (MiniMuffs, Natus Medical Inc., San Carlos, CA, USA) was applied in infants. MRI compatible headphones with active gradient noise cancelation (Optoacoustics Ltd, Moshav Mazor, IL) were used to provide additional gradient noise cancelation and to present the auditory stimulus. All infant data collection was supervised by a clinician (doctor or nurse) and physiological parameters (oxygen saturations, heart and respiratory rate and axillary temperature) were monitored throughout. Adult subjects were studied when awake with suitable hearing protection and the same headphones. These adults were informed about the stimuli types before data collection, but received minimal instruction about the protocol to minimize bias.

Magnetic resonance images were acquired using a 3 Tesla MRI scanner (Philips Achieva, Best, NL) located on the NICU at St Thomas Hospital with a 32-channel receive head coil. High-resolution structural T1-weighted and T2-weighted images were acquired for all subjects studied for image registration purposes (Merchant et al. 2009). BOLD contrast fMRI data were acquired using an echo-planar imaging sequence with TR 1500 ms, TE 45 ms, FA 90°, resolution 2.5 x 2.5 x 3.25 mm^3^ with 0.75 mm slice gap. Adult data were collected with identical parameters except with a decreased spatial resolution of 3.5 x 3.5 x 4.5 mm^3^ (1.45 mm slice gap), so that the same temporal resolution as the neonatal acquisition could be maintained.

### Stimuli and Paradigm

The paradigm consisted of an auditory stimulus played through headphones (operating as the conditioned stimulus) and a precisely controlled passive motor stimulus (operating as the US). Stimulus control, monitoring, and synchronization between stimuli presentation and image acquisition were achieved though custom code developed in the LabVIEW software environment (National Instruments, Austin, TX, USA).

The auditory stimulus consisted of a jingling bell sound previously used in a study with young children (Emberson et al. 2015) played for 6 s at 90–100 dB SPL at the headphone delivery point (with further attenuation by the dental putty and earmuffs of approximately 20–25 dB in neonates (Merchant et al. 2009) and approximately 25 dB in adults with polyurethane earplugs). Sensorimotor stimulation (right wrist flexion/extension in infants and right hand second and third digit flexion/extension in adult subjects) was performed using a pneumatically actuated MRI compatible custom-made robotic device (Allievi et al. 2013). Subjects were first presented with each of the two stimuli independently using a block paradigm [6 s of task (5–6 blocks of passive movement and 5–9 blocks of auditory stimulation) alternated with 24 s of rest] to familiarize them with the stimuli and to map baseline functional responses.

A delay classical conditioning paradigm was then presented following the baseline condition, consisting of the auditory stimulus (6 s) concurrently presented with the passive movement stimulus (5.5 s); with the passive movement commencing 0.5 s later and both terminating together. Each learning epoch consisted of 4–6 paired trials presented every 1.5 s, followed by a period of rest (24 s) and then two randomly ordered test trials (one consisting of the two stimuli presented together and the other consisting of the auditory stimulus alone) separated by a further 24 s (Fig. 1). These auditory alone stimuli served as the CR test condition. The total experiment consisted of 11 epochs lasting a total of 22 minutes.

To further test whether any induced learning was dependent on paired presentation, or could have simply arisen by simple (and not co-occurring) exposure to the two distinct stimuli, we also presented a control paradigm of identical length during which the two stimuli were presented randomly in a nonoverlapping fashion to a small separate group of 4 infants. In this control experiment, subjects were presented with identical auditory and passive movement stimuli as in the learning experiment. The structure and length (22 min) of the paradigm was also identical to the learning paradigm with the exception that only one of the stimuli was presented at a time, resulting in a random sequence of unpaired events (38 stimuli of each type).

### Data Analysis

MRI data were processed using tools implemented in FSL (www.fmrib.ox.ac.uk/fsl) (Woolrich et al. 2009). To reduce the confounding effects of head motion, data sets were cropped to exclude prolonged or excessive motion estimated from a preliminary analysis which identified outlier volumes based on signal intensity (rather than framewise displacement which would not necessarily identify the artifactual effects of motion on our signal of interest). The root mean square intensity difference of volume *N* to volume *N* + 1 (Power et al. 2012) was computed and the image was cropped to preserve the longest possible contiguous section of data before or after sections where either 12 consecutive volumes (more than 18 s of continuous motion) or more than 13% of volumes were corrupted by motion artifact. Residual high signal artifacts due to motion were then further corrected using AFNI 3dDespike (http://afni.nimh.nih.gov/). The included fMRI images were then preprocessed using a pipeline optimized for infant subjects consisting high pass filtering (with 0.01 Hz cut-off frequency) MCFLIRT rigid body motion correction, slice timing correction, brain extraction using BET, and spatial smoothing (Gaussian of FWHM 5 mm) (Dall’Orso et al. 2018). Residual artifacts due to effects such as cardiorespiratory noise and residual head motion were then regressed from the data after identification using independent component analysis (Griffanti et al. 2017).

Lower level functional activation maps were obtained by analyzing the fMRI data using a voxel-wise general linear model (GLM) as implemented in FEAT v6.00, consisting of the experimental paradigm (one explanatory variable representing the occurrence of all auditory stimuli and a second representing the test condition consisting of the sound alone (CR), see Supplementary Fig. S1) convolved with a neonatal-specific hemodynamic response function basis function set (Arichi et al. 2012) and six motion parameters (translation and rotation) derived from the initial rigid body motion correction. This choice of GLM was selected to identify activation related to variance explained by the CR alone without a priori assumption of its shape and/or location. The unthresholded *z*-statistical maps from each subject were then registered to the subject’s own high resolution T2-weighted image and then to a 41 week premenstrual age template brain (Serag et al. 2012) (infants) or the MNI152 space (adults) using nonlinear registration as implemented in FSL FNIRT (Jenkinson et al. 2012). Group level effects were then identified using a nonparametric one-sample *t*-test implemented with permutation methods (FSL randomise v2.0). To reduce false positive rates, this test was optimized using threshold-free cluster enhancement (with variance smoothing (5 mm), height parameter 3.1 and extent 0.4) (family-wise error corrected *P* < 0.01 two-tailed) (Nichols and Hayasaka 2003). The percentage signal change (relative to the baseline calculated from the 6 s preceding stimulus onset) associated with different conditions was calculated from the primary sensorimotor cortex within a binarized mask (SM1-mask) derived from the group results from the baseline wrist response (generated with subjects of both learning and control groups prior to the learning (or control) paradigm) to avoid bias towards either of the specific group. Considering that negative BOLD responses are occasionally seen in both adults and infants, with several groups suggesting different explanations including global modulation of response amplitude, oxygen extraction that exceeds local supply, and neural inhibition (Mullinger et al. 2014; Mayhew et al. 2016), we looked at the absolute change in amplitude rather than its direction. Differences in the amplitude of the BOLD response within the common mask were then tested at a group level using statistical tests as implemented in the statistical toolbox of Matlab R2017b (The Mathworks Inc., Natick, MA, USA).

Position data from a fiber optic goniometer fitted on the robotic interface were investigated in the adult group to explore whether there was a behavioral CR. For each subject (*n* = 9, data from one subject were lost), the angular position was extracted for the two test trials: one where the device was moved and the other not (11 trials each condition). The displacement of each pair of trials was then normalized to the maximum amplitude of the condition when movement was induced. The timeseries of the average position across trials and subjects (*n* = 99) was then computed (see Supplementary Fig. S3). A similar analysis could not be performed in the infant group because they do not have the strength required to overcome the friction of the device and actively move the handlebar.

## Results

Task fMRI data were successfully acquired in a group of 17 healthy infants during presumed natural sleep (median post menstrual age at scan: 40 weeks +1 days, range: 38 + 3 to 42 + 3 weeks). Data from 4 infants were discarded entirely due to severe head motion throughout data collection, unexpected brain injuries (large birth related intracerebral hemorrhage) identified on the anatomical images or equipment failure. Data from the learning experiment alone were discarded or not collected in additional 7 infants due to them waking and/or moving excessively during that specific part of the experiment. The final infant learning and control study groups consisted respectively of 13 infants (median gestational age at birth 38 weeks + 4 days, range: 32 weeks + 2 days to 41 weeks + 6 days) scanned at term equivalent age (median post menstrual age 40 weeks + 5 days, range: 38 weeks + 3 days to 42 weeks + 1 days), and 4 infants (median gestational age at birth: 38 weeks + 1 days, range: 30 weeks + 4 days to 39 weeks + 2 days) scanned at term equivalent age (median post menstrual age 40 weeks + 6 days, range: 39 weeks + 3 days to 42 weeks + 3 days), all of whom were healthy at the time of scanning. The adult study group comprised 10 healthy adult volunteers (6 females, 4 males, age range: 25–39-years old).

**Figure 2:**
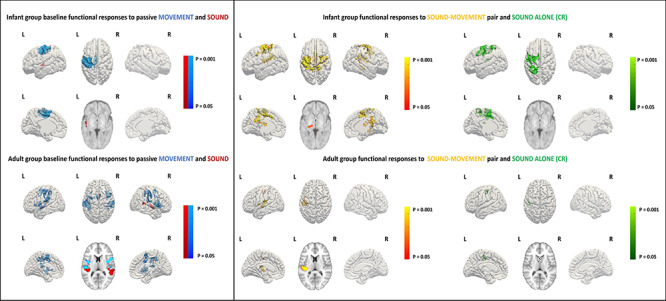
FMRI group results of baseline (left) and learning (right) sequences. Significant clusters of functional response are projected onto the surface of an age specific 3D brain template; infant group maps are shown on the top row (*n* = 11 baseline sound, *n* = 13 baseline movement and learning) and adult group maps (*n* = 10 baselines and learning) are shown on the bottom row. Group maps of the baseline functional response to passive hand/fingers movement are shown in blue with localization to the contralateral SM1 for infants and also in the ipsilateral S1 and SMA for adults. Group maps of the baseline functional response to sound are shown in red, with localization to the left auditory cortex for both infants and adults. In red–yellow are shown the group maps during the learning condition (coupled sound and movement), which cover the areas activated during the baseline as well as additional activation in the midline SMA. The CR to the sound alone condition is shown in green, showing additional activity in the contralateral SM1 for infants and S1 for adults.

**Figure 3:**
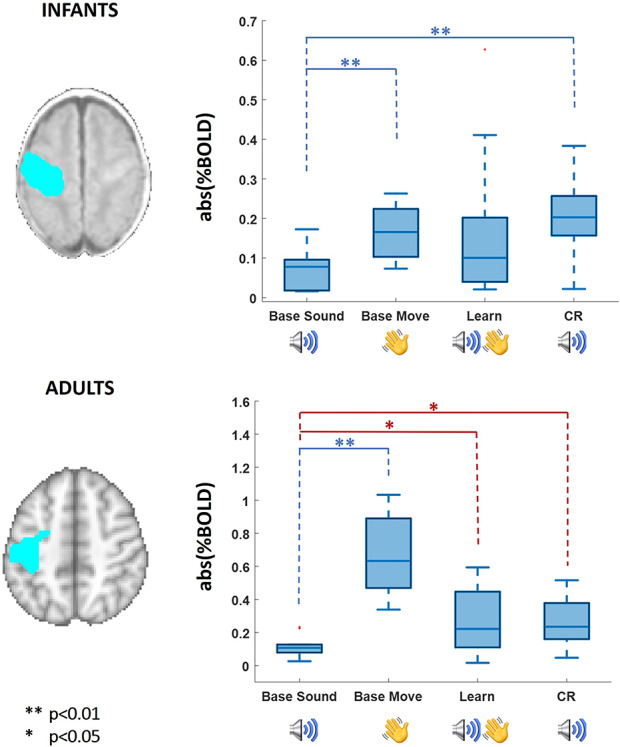
Measure of BOLD percentage signal change in SM1 calculated within a mask (blue) derived from the response to the passive movement baseline response. The absolute value of the percent signal change was calculated for the sound and movement prelearning (Base, *n* = 11, *n* = 13), and learning blocks (Learn, *n* = 13) and sound alone (CR, *n* = 13). On the top box-plot, infants’ data show a significant difference of response between the sound prelearning and both prelearning hand movement and sound alone during learning. On the bottom box-plot, adult data show a significant difference between the amplitude of BOLD responses within the SM1 mask in the baseline sound and prelearning hand movement (blue, Friedman test applied to all conditions), and baseline sound to both learning conditions (paired stimuli and CR) (red, Wilcoxon signed rank test).

**Figure 4:**
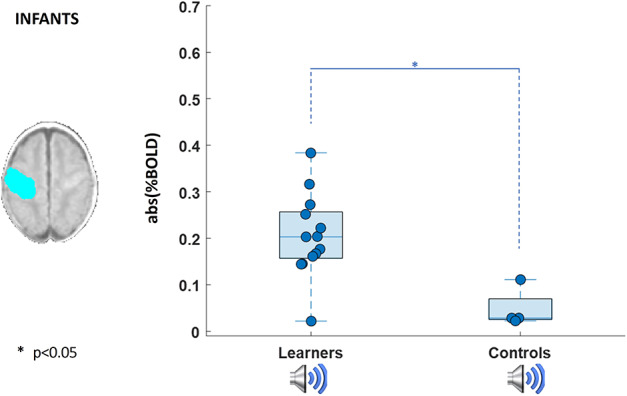
Measure of BOLD percentage signal in SM1 calculated within a mask (blue) derived from the area of response to the passive movement baseline response. Absolute value of percent signal change was calculated for the sound response in the learning group *n* (*n* = 13) and control group (*n* = 4).

In the baseline condition, significant clusters of positive BOLD functional activity were identified in the primary auditory cortices following the auditory stimulus; and in the contralateral (left) primary sensorimotor cortex (SM1) in response to passive motor stimulation. As expected, when both stimulus types were presented concurrently during the learning condition, functional activation was detected within both the primary auditory cortices and contralateral SM1 corresponding to the baseline condition, with additional activity observed within the ipsilateral SM1 and supplementary motor area (SMA). There was no significant relationship between the patterns of activity in any of the conditions and GA at birth across our infant subjects. Most importantly however, and consistent with associative learning having taken place, infants were found to exhibit a significant CR to the auditory alone test condition in the contralateral SM1 and anteriorly in the premotor cortex (PMC) (*P* < 0.01, Kruskal–Wallis test, Fig. 2 top row). In keeping with the spatial representation of this activity, the amplitude of the BOLD response within the identified SM1-mask cluster was significantly higher when the wrist was actually moved during the baseline passive movement and following learning in response to the auditory alone test condition in comparison with the baseline (prelearning) auditory condition (*P* < 0.01, Kruskal–Wallis test, Fig. 3 top row). This increase in activity was not seen in the subset of 4 infants who received the control paradigm of nonoverlapping stimuli (*P* < 0.05, Wilcoxon rank sum test, Fig. 4; group activation patterns in the control group are included in Supplementary Fig. S2).

As with the infant subjects, the adult response during learning could be localized to the left auditory cortex, contralateral SM1 and SMA, all of which were engaged during the respective auditory and passive movement baseline conditions (Fig. 2 bottom row). When comparing all conditions simultaneously (Friedman test), as expected, BOLD responses within the SM1 mask were of significantly higher amplitude in the baseline passive movement condition in comparison with a negligible response in the baseline auditory condition (Fig. 3 bottom row). BOLD signal change was also significantly higher within SM1 during both the learning condition (both stimuli) and in the CR compared with the baseline auditory condition when tested individually (Wilcoxon signed rank test, *P* = 0.0371 and *P* = 0.0195, respectively). Although we measured a cortical response to the auditory alone test condition, we did not see any evidence of the CR being associated with any behavioral response (i.e., actual finger movement) in our adult subjects (see Supplementary Fig. S2).

## Discussion

Using a classical conditioning auditory-sensorimotor paradigm and fMRI, we have demonstrated that the early human brain is already capable of processing external and simultaneous multisensory information within the two distinct primary sensory cortices. We further found that this multisensory stimulus presentation can directly influence cortical activity in a crossmodal manner, as evidenced by a CR in the primary sensorimotor cortex, even in the absence of its archetypal substrate. Lastly, we also found that the process of encoding information during associative learning in the neonatal period engages wider cortical association regions across relevant neural networks.

In both infants and adult volunteers, we identified a CR response within the primary sensorimotor cortices as a result of our simple auditory-sensorimotor conditioning paradigm. Learning-related changes in activity in the mature motor/premotor cortex have also been previously described following implicit learning of a simple motor sequence or classical conditioning (Hazeltine et al. 1997; Ramnani et al. 2000). However, as shown in previous studies, this change in neuronal activity was not accompanied by a measurable behavioral CR in our adult subjects (Landau et al. 2004; Kelly and Garavan 2005; McNealy et al. 2006). Therefore, the identified increase in BOLD activity could be explained by induced increase in neuronal excitability, or more likely represents a predicted execution of movement and its sensory consequence (Ramnani et al. 2000; Kilteni et al. 2018). In favor of the latter explanation, motor imagery activates the same somatotopic area that would be engaged in the production of the intended movement (Ehrsson et al. 2003) and the CR in our infant subjects included activity in the contralateral PMC, which is an area thought to play a key role in planning and preparing for movements (Halsband et al. 1993). In this case, the sensorimotor CR would be part of an internally generated forward model which would represent the internal simulation of a movement and predicts the motor outcome including the resulting sensory feedback (Kilteni et al. 2018). Such a forward model would be created and updated based on the spatiotemporal features of previous experience; therefore, the repeated pairing of the auditory cue prior to the movement would reinforce prediction of upcoming movement. This sensory prediction then enables fast appropriate reactions and adaptation as sensory feedback is intrinsically noisy and delayed in nature (Wolpert and Flanagan 2001; Kilteni et al. 2018). Although these models are likely refined throughout life for environmental adaptation and support learning, this mechanism is of particular relevance during early development as sensory and motor frameworks are rapidly maturing (Dooley and Blumberg 2018). Although an alternative but related explanation is that the identified activity could represent prediction error, we did not see activity in regions such as the anterior cingulate or prefrontal cortices as is typically seen in this context (Holroyd et al. 2004; Garrison et al. 2013).

Our results identified the primary sensorimotor cortices as the encoders of our associative learning task, supporting the idea that they are not only involved in basic sensory perception but also higher level processes such as multisensory integration and experience specific memory encoding (Zhou and Fuster 2000; Weinberger 2004, 2011; Schroeder and Foxe 2005; Headley and Weinberger 2015). This is in agreement with studies in mature mammals, which demonstrated associative learning induced crossmodal activation in the primary sensory cortical networks responsible for processing tactile information following visual-tactile conditioning (Zhou and Fuster 2000); in the primary visual cortex in response to sound following auditory–visual conditioning (Headley and Weinberger 2015); and in the auditory cortex in response to visual and sensorimotor stimulation in a multimodal task (Brosch et al. 2005). Brosch et al. further suggested that the observed nonauditory neuronal firing in the auditory cortex may have indicated expectation or preparation of the predicted sound as a result of the learnt association between the stimuli of different modalities. Importantly, this also demonstrates that multimodal association in the neonatal and adult brain can also be made between two neutral stimuli in the absence of a reinforcer, and thus without the need of strong motivational drivers.

Although changes in cortical activity were seen in both of our subject groups, there were significant differences in their behavioral state during the experiment; as the adults were studied during their typical awake state, while the infants were studied during sleep which also corresponds to their typical state (as they spend 16–18 h per day asleep; So et al. 2007). This may either suggest that this form of cortical processing is an intrinsic human property regardless of behavioral state, or rather may reflect a clear difference between the distinct developmental stages of the two populations and their need to extrapolate ongoing information about everyday sensory experience during their typical but different behavioral states. In keeping with the latter, mismatch negativity (MMN) studies have found attenuated cortical responses to variation in stimulus pattern in sleeping adults, while a large amplitude MMN response is observed in both sleeping and awake infants (Sallinen et al. 1994). Our findings therefore endorse further studies to elucidate precisely how learning occurs in different states and developmental stages. Sleep in particular, is thought to play a key role in memory consolidation through mediating early neuroplasticity as it facilitates fundamental processes such as synapse formation and pruning, both of which are impaired by early sleep deprivation (Shaffery et al. 2006). These effects appear to be further modulated by sleep state, with more significant learning seemingly occurring during deeper sleep stages (Tarullo et al. 2011, 2016). It would be of great interest in future studies to understand how it may have contributed to our results.

In addition to a CR in our infant subjects, the learning task induced a widespread pattern of activation across the entire sensorimotor network including the ipsilateral SM1 and the SMA (Fig. 2, top row middle). Although a direct quantitative comparison is not possible due to the different amounts of data across conditions and the experimental design, this qualitative evidence is consistent with data from adults showing a spatial increase in motor cortical representation during the encoding phase of a motor learning task, suggesting that the entire motor network contributes to the learning process itself (Pascual-Leone et al. 1994; Hardwick et al. 2013). The involvement of the SMA during learning in our infant subjects is of particular interest, as it is commonly observed during implicit motor learning tasks in adults where it is thought to encode and coordinate temporal sequences of motor behavior through generating an internal predictive model (Hazeltine et al. 1997; Sanes 2003). Increasing activity within the SMA in sensorimotor functional responses is seen with increasing postnatal and postmenstrual age across the human preterm period (Allievi et al. 2016), which further suggests that ex-utero sensorimotor experience may drive the establishment of SMA coactivation and connectivity in early life.

Activity across the sensorimotor network during learning is of particular significance, as learning can result in sustained changes in resting functional connectivity between coactivated regions in a task, in keeping with network specific neuroplasticity (Pascual-Leone et al. 1994; Büchel et al. 1999; Sanes 2003; Albert et al. 2009). Both the timing and strength of learning-induced changes in connectivity significantly correlate with task performance, suggesting that increased interaction between involved brain regions is a crucial neural mechanism underlying associative learning (Büchel et al. 1999; van den Bos et al. 2012). Sensory experience and learning can also directly modify cortical organization, as it has previously been demonstrated that body map enlargement can occur as a result of classical conditioning (Siucinska and Kossut 2004; Galvez 2006; Chau et al. 2014). This is of particular relevance in the developing human brain, as long-range functional connectivity measures increase and associated network structures rapidly mature during the crucial perinatal period and through early infancy (Doria et al. 2010; Thomason et al. 2013; Gao et al. 2015). In this context, our results suggest that associative learning may play a fundamental role in this process through triggering and modulating ongoing patterns of relevant network connectivity.

Although the study protocol was successful in achieving the aims of the study, we acknowledge limitations which could be considered or addressed in future studies. The auditory stimulus presented several inherent challenges due to the loud MRI scanner environment and the resultant need to reduce ambient noise. Although we were able to carefully control and monitor the range of dB delivered by the headphones, the actual level of sound at the ear may have varied between subjects and is likely to have been different in the adult and infant groups due to the different hearing protection applied. Together, this may have introduced intersubject variability in the pattern of auditory responses in the neonates which may explain their unilateral nature. We also cannot rule out that the lack of a bilateral auditory response and/or activation in areas such as the anterior cingulate and prefrontal cortices may have arisen from our small sample size leading to insufficient power to detect small effect sizes.

Although our results suggest that there are several similarities between infant and adult brain activity during multimodal associative learning, they also hint towards subtle differences between groups both in the spatial pattern and amplitude of the responses. In addition to quantifying these effects in more detail in dedicated studies, it would also be of great interest to explore other relevant phenomena including how stimulus characteristics (such as frequency and amplitude) are encoded during this process. Although our infant group was carefully screened to include only those who were healthy at the time of study and had no significant brain injuries, it did include some infants born preterm who by nature of their neonatal course would have had different sensory experience to those born at full term. Therefore, although our findings are robust across a slightly heterogenous population, a next important step will also be to understand whether our results might differ for infant populations, behavioral states, and stimulus types through replicating our experiment with these conditions.

## Conclusion

Our results demonstrate that the developing human cortex is not only able to process multimodal sensory information, but can also additionally alter its activity to learn associations between distinct stimulus types. Moreover, the encoding of this process was found to engage additional associative regions which comprise the relevant wider neural network. This implies that even shortly after birth, activity and network connections in the developing brain are being constantly shaped by the environment, with wide-reaching implications for understanding the neural processes which underlie early human development and the alterations which may lead to later neurodevelopmental disorders including speech and language disorders.

## Notes

The authors thank Emer Hughes, Elaine Green, Joanna Allsop, Katie Colford, and Louise Dillon who were all involved in the acquisition of the data. The authors also thank the clinical staff on the neonatal intensive care unit at St Thomas Hospital London for supporting the work and the parents who consented for their infants to participate in the work. *Conflicts of Interest*: The authors have no conflicts of interest to disclose.

## Funding

Engineering and Physical Sciences Research Council (grant EP/L016737/1 to S.D.) and in part by the Chalmers University of Technology Area of Advance in Life Science Engineering (to S.D.). Data acquisition and analysis were funded through a Medical Research Council (MRC) Clinician Scientist Fellowship (MR/P008712/1 to T.A. and T.P.). Device development were supported in part by the European Commission (grants H2020 ICT-644727 COGIMON, FETOPEN-829186 PH-CODING, MSCA-ITN 861166 INTUITIVE to E.B.), as well as by the UK Engineering and Physical Sciences Research Council (EPSRC) MOTION (grant EP/NO29003/1 to E.B.). The authors additionally acknowledge support from the Department of Health via the National Institute for Health Research (NIHR) comprehensive Biomedical Research Centre award to Guy’s & St Thomas’ NHS Foundation Trust in partnership with King’s College London and King’s College Hospital NHS Foundation Trust, and the Wellcome Engineering and Physical Sciences Research Council (EPSRC) Centre for Medical Engineering at Kings College London (WT 203148/Z/16/Z).

## Author Contributions

S.D.O. and T.A. were involved in all aspects of the study including study design, data collection, data analysis and interpretation, and manuscript preparation. K.V., J.B., and C.O. were involved in subject recruitment and data collection. W.P.F., P.D.B, A.D.E., and E.B. were involved in study design and data interpretation. All authors reviewed and contributed to manuscript preparation.

## Supplementary Material

Supplementary_material_bhaa340
