# Supplementary material for: Cortical Processing of Multimodal Sensory Learning in Human Neonates
Source: Cereb Cortex. 2020 Nov 18;31(3):1827–36. doi: 10.1093/cercor/bhaa340 (PMC7869081; doi:10.1093/cercor/bhaa340)
Supplement: Supplementary_material_bhaa340 [file supplementary_material_bhaa340.docx]

**Supplementary figure**

**
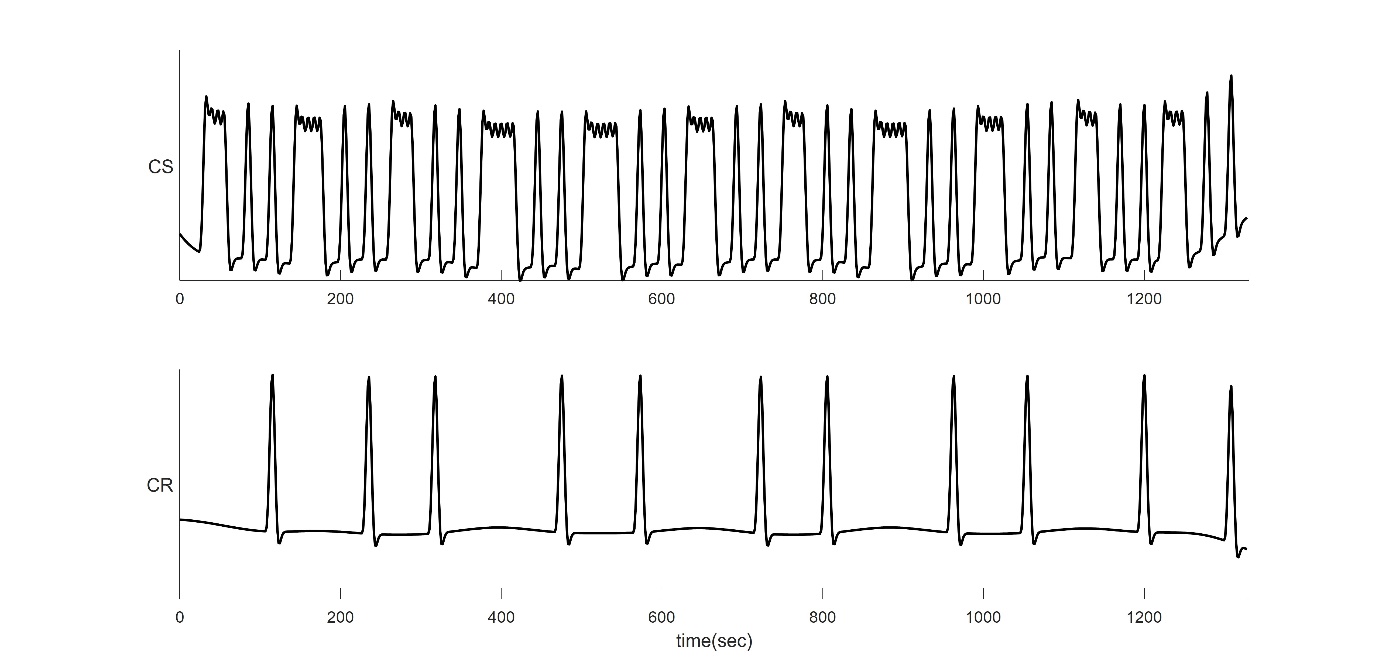
**

Figure S1: Representation of the GLM used for the low-level statistical analysis. The first regressor represents the auditory stimuli occurrence (conditioned stimulus, CS) whereas the second regressor, which is the main focus of the analysis, represent the conditioned response test trials (CR). In addition, the temporal and dispersion derivatives as well as the 6 motion parameters were added to the final model for analysis.

.
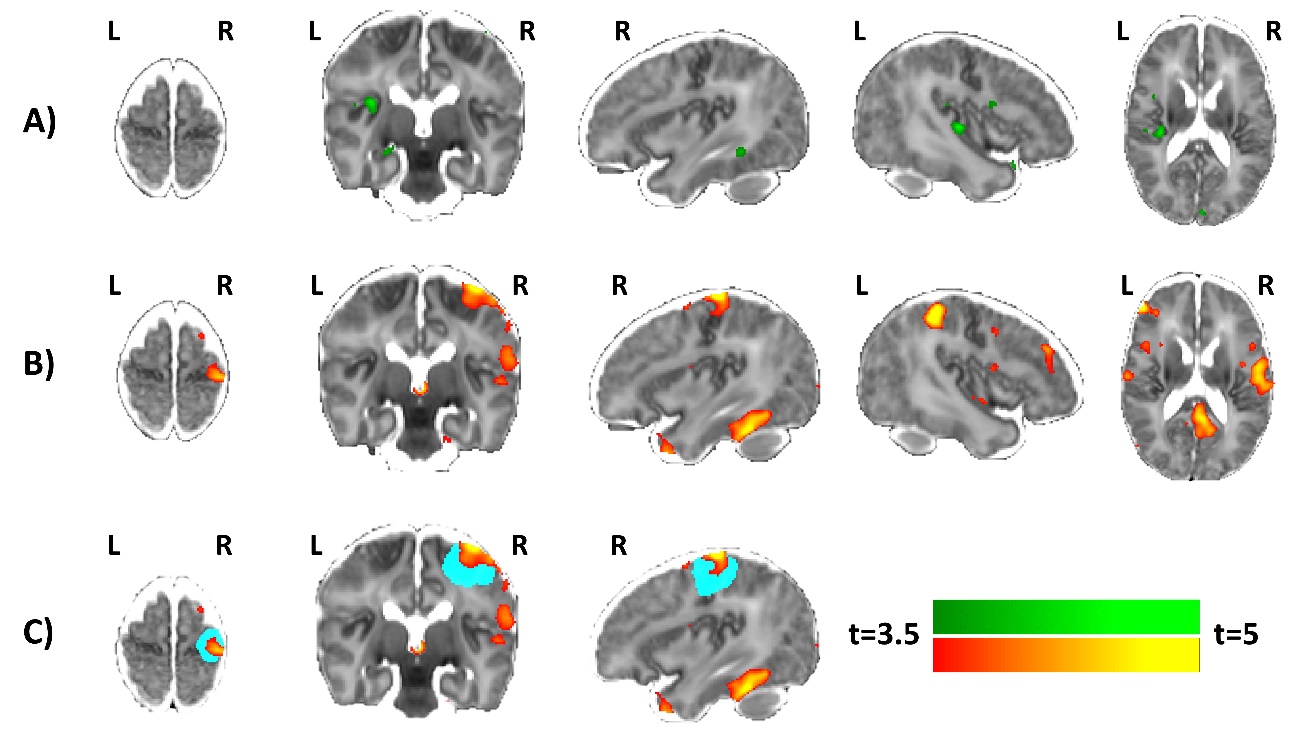


Figure S2: Group results of infant control group (n=4). A) Functional map in response to the auditory stimulation during the control condition. B) Functional map in response to the sensorimotor stimulation during the control condition. C) Functional map in response to the sensorimotor stimulation during the control condition overlapping onto the sensorimotor mask used for the BOLD calculation.


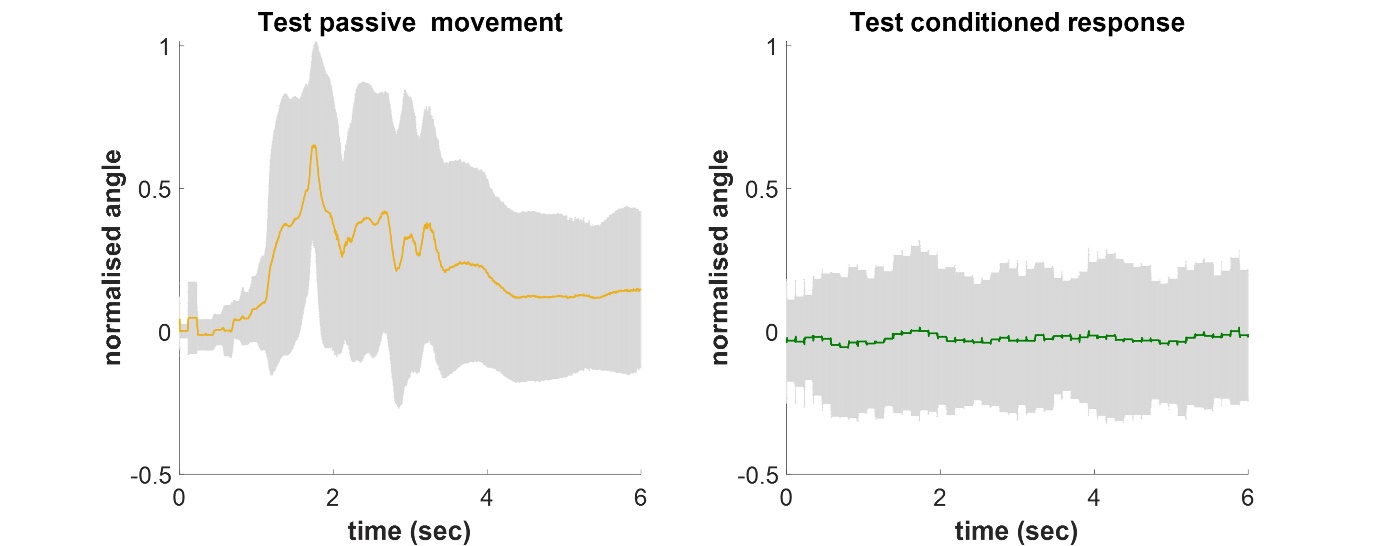


Figure S3: Normalised average flexion/extension angle at the metacarpophalangeal joint of 2^nd^ and 3^rd^ fingers during 6 seconds trials in two conditions: test passive movement (left, yellow) and test conditioned response (right, green). Average and standard deviation calculated from 99 trials (9 subjects and 11 trials per condition). During the test conditioned response there is no evidence of active movement from the subject in response to the learnt association between the auditory cue and passive movement.
